# Benefits and harms of implementing [^18^F]FDG-PET/CT for diagnosing recurrent breast cancer: a prospective clinical study

**DOI:** 10.1186/s13550-021-00833-3

**Published:** 2021-09-22

**Authors:** Marianne Vogsen, Jeanette Dupont Jensen, Oke Gerke, Anne Marie Bak Jylling, Jon Thor Asmussen, Ivar Yannick Christensen, Poul-Erik Braad, Peter Thye-Rønn, Katrine Lydolph Søe, Marianne Ewertz, Malene Grubbe Hildebrandt

**Affiliations:** 1 Department of Oncology, Odense University Hospital, 5000 Odense C, Denmark; 2 Department of Nuclear Medicine, Odense University Hospital, Odense, Denmark; 3 Department of Clinical Research, University of Southern Denmark, Odense, Denmark; 4 OPEN, Odense Patient Data Explorative Network, Odense University Hospital, Odense, Denmark; 5 Center for Personalized Response Monitoring in Oncology (PREMIO), Odense University Hospital, Odense, Denmark; 6 Department of Pathology, Odense University Hospital, Odense, Denmark; 7 Department of Radiology, Odense University Hospital, Odense, Denmark; 8 Diagnostic Center, Department of Medicine, Odense University Hospital, Svendborg, Denmark; 9 Department of Breast Surgery, Odense University Hospital, Odense, Denmark; 10 Center for Innovative Medical Technology, Odense University Hospital, Odense, Denmark

**Keywords:** Recurrent breast cancer, [^18^F]FDG-PET/CT, Accuracy, Distant metastases, Incidental findings

## Abstract

**Background:**

[^18^F]-fluorodeoxyglucose-positron emission tomography/computed tomography ([^18^F]FDG-PET/CT) has been implemented sporadically in hospital settings as the standard of care examination for recurrent breast cancer. We aimed to explore the clinical impact of implementing [^18^F]FDG-PET/CT for patients with clinically suspected recurrent breast cancer and validate the diagnostic accuracy.

**Methods:**

Women with suspected distant recurrent breast cancer were prospectively enrolled in the study between September 2017 and August 2019. [^18^F]FDG-PET/CT was performed, and the appearance of incidental benign and malignant findings was registered. Additional examinations, complications, and the final diagnosis were registered to reflect the clinical consequence of such findings. The diagnostic accuracy of [^18^F]FDG-PET/CT as a stand-alone examination was analyzed. Biopsy and follow-up were used as a reference standard.

**Results:**

[^18^F]FDG-PET/CT reported breast cancer metastases in 72 of 225 women (32.0%), and metastases were verified by biopsy in 52 (52/225, 23.1%). Prior probability and posterior probability of a positive test for suspected metastatic cancer and incidental malignancies were 27%/85% and 4%/20%, respectively. Suspected malignant incidental findings were reported in 46 patients (46/225, 20.4%), leading to further examinations and final detection of nine synchronous cancers (9/225, 4.0%). These cancers originated from the lung, thyroid, skin, pancreas, peritoneum, breast, kidney, one was malignant melanoma, and one was hematological cancer. False-positive incidental malignant findings were examined in 37/225 patients (16.4%), mainly in the colon (*n* = 12) and thyroid gland (*n* = 12). Ten incidental findings suspicious for benign disease were suggested by [^18^F]FDG-PET/CT, and further examinations resulted in the detection of three benign conditions requiring treatment. Sensitivity, specificity, and AUC-ROC for diagnosing distant metastases were 1.00 (0.93–1.0), 0.88 (0.82–0.92), and 0.98 (95% CI 0.97–0.99), respectively.

**Conclusion:**

[^18^F]FDG-PET/CT provided a high posterior probability of positive test, and a negative test was able to rule out distant metastases in women with clinically suspected recurrent breast cancer. One-fifth of patients examined for incidental findings detected on [^18^F]FDG-PET/CT were diagnosed with clinically relevant conditions. Further examinations of false-positive incidental findings in one of six women should be weighed against the high accuracy for diagnosing metastatic breast cancer.

*Trial registration* Clinical.Trials.gov. NCT03358589. Registered 30 November 2017—Retrospectively registered, http://www.ClinicalTrials.gov

**Supplementary Information:**

The online version contains supplementary material available at 10.1186/s13550-021-00833-3.

## Background

Despite improved treatment for primary breast cancer, distant metastases will occur in 20–30% of patients. Metastatic breast cancer (MBC) has a poor prognosis with a five-year survival of 25% [1, 2]. Detection of distant metastases is important for treatment decisions and may impact the prognosis of the disease [3]. The accuracy of diagnostic modalities for diagnosing distant recurrent breast cancer varies, but [^18^F]-fluorodeoxyglucose-positron emission tomography/computed tomography ([^18^F]FDG-PET/CT) has higher accuracy than conventional methods [4–6]. However, no clear recommendations are given in current clinical guidelines on diagnostic modality when examining patients with a clinical suspicion of recurrent disease [3, 7, 8]. Reasons for this may include that few prospective studies have been conducted, and knowledge is lacking of the clinical impact of using [^18^F]FDG-PET/CT for the examination of women suspected of recurrent breast cancer.

Like in other cancers, incidental findings suspicious for malignancy may be detected by [^18^F]FDG-PET/CT when used to diagnose and to stage cancer [9, 10]. Incidental malignant findings may generate additional examinations, complications, and anxiety, leading to avoidance of using [^18^F]FDG-PET/CT. Data are scarce on the extent and clinical consequences of false-positive incidental findings on [^18^F]FDG-PET/CT in women suspected of recurrent breast cancer.

[^18^F]FDG-PET/CT has already been implemented as the standard of care for diagnosing recurrent breast cancer in our institution [4]. Therefore, this prospective study aimed to explore the clinical impact of performing [^18^F]FDG-PET/CT as a stand-alone examination in patients with clinical suspicion of recurrent breast cancer. In addition, we aimed to validate the accuracy of [^18^F]FDG-PET/CT for diagnosing distant metastases.

## Methods

This clinical study was conducted at Odense University Hospital, Denmark, from September 1, 2017, to August 31, 2019. We used a prospective diagnostic design to validate the diagnostic accuracy of [^18^F]FDG-PET/CT for diagnosing recurrent breast cancer according to the STARD guideline [11]. Women were referred for [^18^F]FDG-PET/CT on suspicion of recurrent breast cancer. The analyses comprised the detection of incidental findings by [^18^F]FDG-PET/CT, the clinical impact of further examinations and diagnosis of concurrent diseases, and the diagnostic accuracy of diagnosing distant metastasis.

The study was approved by the Danish Ethics Regional Committee (S-20170019), and all subjects signed an informed consent form. The secure systems Research Electronic Data Capture (RedCap) and SharePoint were used for data storing and data management. The study was conducted following the Declaration of Helsinki and registered at Clinical.Trials.gov (NCT03358589).

### Patients

Women were eligible if they were 18 years or older, signed a consent statement, and had a prior diagnosis of early-stage breast cancer. They were identified from symptoms of first distant metastases (e.g., bone pain, fatigue, weight loss, etc., Table 1) or a biopsy-verified local recurrence. For the latter group, all patients were examined by [^18^F]FDG-PET/CT to rule out distant metastases as a standard of care procedure before surgery or medical treatment. Women were excluded from the study if they were pregnant, were treated for other invasive cancers at the time of inclusion, or suffered from other conditions that interfered with the patients' understanding of the study. Women with biopsy-verified MBC before referral to [^18^F]FDG-PET/CT were not eligible.

**Table 1:** Symptoms leading to referral for [^18^F]FDG-PET/CT in 225 women suspected of recurrent breast cancer

Symptoms	All patients	Metastatic breast cancer
	*n* (%)	*n* (%)
Bone pain	90 (40.0)	24 (46.2)
Local recurrence examination	45 (20.0)	9 (17.3)
Fatigue	33 (14.7)	5 (9.62)
Weight loss	27 (11.9)	1 (1.92)
Abnormal blood test^†^	25 (11.1)	5 (9.62)
Abdominal discomfort	23 (10.2)	7 (13.5)
Palpable lymph node	16 (7.11)	7 (13.5)
Shortness of breath	11 (4.89)	6 (11.5)
Changes in skin	7 (3.11)	3 (5.77)
Nausea and/or vomiting	7 (3.11)	0 (0.00)
Other^‡^	90 (40.0)	12 (23.1)
Total	225 (100)	52 (100)

^†^Elevated liver enzymes, anemia, or other blood tests

^‡^Pain elsewhere, headache, medullary stress, pleural fluid, detected randomly in radiologic tests, or other symptoms

### Data collection

Patient- and disease-specific data comprised reason for referral and referring department, age, time from primary breast cancer to the diagnosis of MBC, [^18^F]FDG-PET/CT and magnetic resonance imaging (MRI) scan reports, pathology, and medical records.

We made the following definitions: (1) When there was more than one prior breast cancer diagnosis, we used the first breast cancer diagnosis to calculate the time from primary to recurrent disease. (2) For multifocal or synchronous bilateral disease, we selected the tumor with the poorest prognostic factors for analysis, (3) lymph node metastases in the axilla were defined as macro-metastases (≥ 2 mm).

### ***[***^***18***^***F]FDG-PET/CT image technique***

PET imaging from the top skull to mid-thigh was performed 60 ± 5 min p.i. with intravenous injection of 4 MBq [^18^F]FDG per kg bodyweight. Blood sugar levels were measured routinely with an upper threshold of 10 mmol/L, and patients fasted at least four hours before [^18^F]FDG injection. All scans were performed on GE Discovery MI 4-ring PET/CT (DMI, GE Healthcare, Waukesha, WI, USA) and GE Discovery 710 PET/CT (DMI, GE Healthcare, Waukesha, WI, USA) scanners following standard guidelines from the European Association of Nuclear Medicine [12]. PET scans were performed using a standard whole-body acquisition protocol with slice overlaps of 40% (DMI) and 25% (D710) and acquisition times of 1½ min (DMI) and 2½ min (D710) per bed position, respectively. The scan field of view was 70 cm. PET data sets were reconstructed using time-of-flight 3D OSEM (GE VPFX, 4 iterations, 17 subsets) with point-spread-blurring correction (GE SharpIR) and using BSREM (GE Q.Clear) with a β penalizing factor of 500) in matrix sizes of 256 × 256 (pixel size 2.74 mm). Corrections for attenuation, randoms, deadtime, and normalization were done inside the iterative loop. Attenuation correction was based on a preceding diagnostic helical scan with in vivo contrast (ultravist 370 I/ml) using a CT protocol with a scan field-of-view (FOV) of 70 cm, 120 kVp, pitch = 0.984, and GE automatic exposure control (GE smartmA: 80–400 mA, NI: 25). CT data were reconstructed in a matrix size of 512 × 512 (pixel size 0.98 mm) and a slice thickness of 3.75 mm. Data for attenuation correction were reconstructed with a Q.AC reconstruction kernel (GE Healthcare, WI, USA) in a FOV of 70 cm, whereas diagnostic images were reconstructed with a GE standard filter in a FOV of 50 cm.

### Reference standard

Biopsies from suitable metastatic lesions served as references and were sent to standard diagnostic procedures, including immunohistochemistry for biomarkers (ER, HER2) [13, 14]. In a few cases with strong clinical confidence in distant metastases, only the biopsy from local recurrences was obtained. If metastatic lesions were detected on [^18^F]FDG-PET/CT but could not be verified by biopsy, patients were followed by imaging according to the location of the lesion. No patients were diagnosed without biopsy verification. Patients with no signs of MBC on [^18^F]FDG-PET/CT were followed for six months by medical records to detect false negatives. False-negative was defined if distant recurrence was revealed within six months from the [^18^F]FDG-PET/CT scan. A patient could have more than one [^18^F]FDG-PET/CT scan during the study period due to a new referral or uncertainty after the initial scan. However, only the first scan was used for accuracy assessment and the remaining for follow-up.

In some cases, MRI was used in addition to biopsy as a follow-up to confirm the absence of metastases, typically in patients with suspected bone metastases. No patients were diagnosed with MBC by MRI alone.

### Outcome measures

A physician in oncology (MV) registered the appearance of incidental benign and malignant findings on [^18^F]FDG-PET/CT to reflect the daily clinical practice of interpreting the scan report. Additional examinations, complications, and the final diagnosis were registered to address the clinical consequence of incidental findings.

For diagnosis of distant metastases, all scans were assessed visually by senior physicians in nuclear medicine and radiology, respectively. A combined image interpretation was used for clinical follow-up, and multidisciplinary conferences were attended in cases of uncertainty. For research purposes to reflect the daily clinical practice of interpreting the scan report, a physician in oncology (MV) ranked the image interpretations on a 5-point Likert scale into one of the following: 0—"no metastatic lesions", 1—“assumingly no metastatic lesions,” 2—“lesion(s) could as well be benign or malignant,” 3—“suspected metastatic lesion(s),” and 4—“highly suspected metastatic lesion(s).” The CE-CT scans were assessed retrospectively by an experienced radiologist, who ranked the CE-CT imaged on the same Likert scale. The radiologist was blinded to [^18^F]FDG-PET/CT images and scan reports but had access to bookmarks used for evaluations of [^18^F]FDG-PET/CT in clinical routine and knowledge of potential subsequent scans for each patient.

Area-under-the-receiver operating curve (AUC-ROC) for diagnosing distant recurrence was performed. For estimates of sensitivity, specificity, positive predictive value, negative predictive value, and accuracy, the 5-point Likert scale was dichotomized into “no metastatic lesions” or “suspected metastatic lesions,” using a cutoff between 2 and 3.

### Statistical analysis

Descriptive statistics are presented according to data type, i.e., categorical variables as frequencies and respective percentages.

Prior and posterior probability for MBC, any metastatic cancer, and suspected incidental malignancies were calculated. Prior probability was calculated by the sum of true positives and false negatives divided by the total number of patients (prevalence). The posterior probability of a positive test was calculated by dividing the true positives by the sum of true positives and false positives (positive predictive value). Likewise, the posterior probability of a negative test was calculated by the false negatives divided by the sum of false negatives and true negatives (1 - negative predictive value).

Point estimates for AUC-ROC and accuracy parameters of dichotomized data (sensitivity, specificity, etc.) were supplemented by 95% confidence intervals (95% CIs). Analyses were performed using Stata/IC 15.0 (StataCorp, College Station, Texas 77845, USA).

## Results

**Fig. 1:**
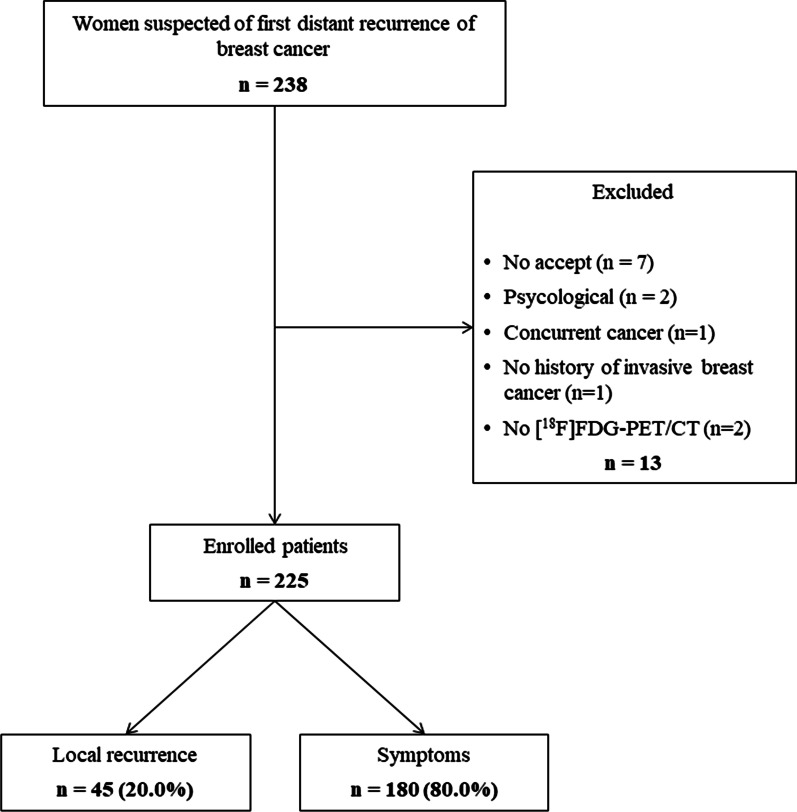
Flowchart of 238 women referred for [^18^F]FDG-PET/CT due to suspicion of first distant recurrent breast cancer, 2017–2019

A total of 238 women were referred for [^18^F]FDG-PET/CT on suspicion of the first distant recurrence of breast cancer. Thirteen patients were excluded for various reasons in Fig. 1, leaving 225 patients eligible for analyses. Reasons for referral included local recurrence in 20% of the patients, while 80% of patients had symptoms such as bone pain, fatigue, or weight loss. The included patients had a median age of 68.0 years (range 33.3–91.2).

Baseline characteristics of the primary breast cancer for the 225 patients appear in Additional file 2: Table 1. Most referred patients (75.6%) received neoadjuvant and/or adjuvant treatment as part of their primary treatment.

Information about symptoms leading to referral is summarized in Table 1. Bone pain was the most common cause for referral in the total cohort and among women diagnosed with MBC. Patients referrals were quite equally distributed between three departments: the Department of Oncology (62/225, 27.6%), the Diagnostic Center (54, 24.0%), and Breast Surgery (46/225, 20.4%), and the remaining patients (63/225, 28.0%) were referred from general practitioners, medical, or other departments.

[^18^F]FDG-PET/CT was performed on 225 women suspected of recurrent breast cancer. Of those, [^18^F]FDG-PET/CT was positive for MBC in 72 patients (32.0%) and negative for MBC (non-MBC) in 153 patients (68.0%). Fifty-two patients (23.1%) were diagnosed with MBC, of whom 48 were verified by distant biopsy and four by local biopsy and strong clinical confidence of distant metastases. The median time from primary breast cancer to the diagnosis of MBC was 8.9 years (range 0.70–38.1), and ten patients (19.2%) experienced distant recurrence while on adjuvant endocrine therapy. Information about metastases and biopsies is presented in Table 2.

**Table 2:** The organ of distant biopsy and the corresponding immunohistochemical profile of metastatic lesions and the distribution of metastatic sites, organ involvement, and burden of the disease appear among 52 women diagnosed with metastatic breast cancer by [^18^F]FDG-PET/CT

Characteristics	Biopsy	[^18^F]FDG-PET/CT
	*n* (%)	*n* (%)
*Organ of biopsy*		
Bone	20 (38.5)	N/A
Lymph node	11 (21.2)	
Liver	7 (13.5)	
Lung and pleural fluid	5 (9.62)	
Breast* (local recurrence)	4 (7.69)	
Other^†^	5 (9.62)	
*ER status*		
Negative (0%)	7 (13.5)	N/A
Positive (1–100%)	43 (82.7)	
Unknown	2 (3.85)	
*HER-2 status*		
Normal	46 (88.5)	
Positive	1 (1.92)	
Unknown	5 (9.62)	
*Location of distant metastases*^*#*^		
Bone	N/A	39 (75.0)
Lung incl. pleural fluid or pleural metastases		30 (57.7)
Lymph node^‡^		28 (53.9)
Liver		16 (30.8)
Other		7 (13.5)
Skin/subcutis		3 (5.78)
CNS		3 (5.78)
*Organ involvement*		
Bone-only^§^	N/A	15 (28.8)
Visceral		16 (30.8)
Lymph node only		2 (3.85)
Mixed		19 (36.5)
*Number of distant metastases*		
1	N/A	0 (0.00)
2–4		8 (15.4)
≥ 5		44 (84.6)
Total	52 (100)	52 (100)

*ER* estrogen receptor, *HER-2* human epidermal growth factor receptor 2

^*^Four patients with distant recurrence were verified by local biopsy and strong clinical confidence of distant metastases

^#^Each patient could have more than one location of metastases

^†^Skin, brain, and other organs

^‡^Loco-regional lymph nodes, mediastinal, or other locations

^§^Including breast and/or axillary lymph nodes

### Clinical impact

**Fig. 2:**
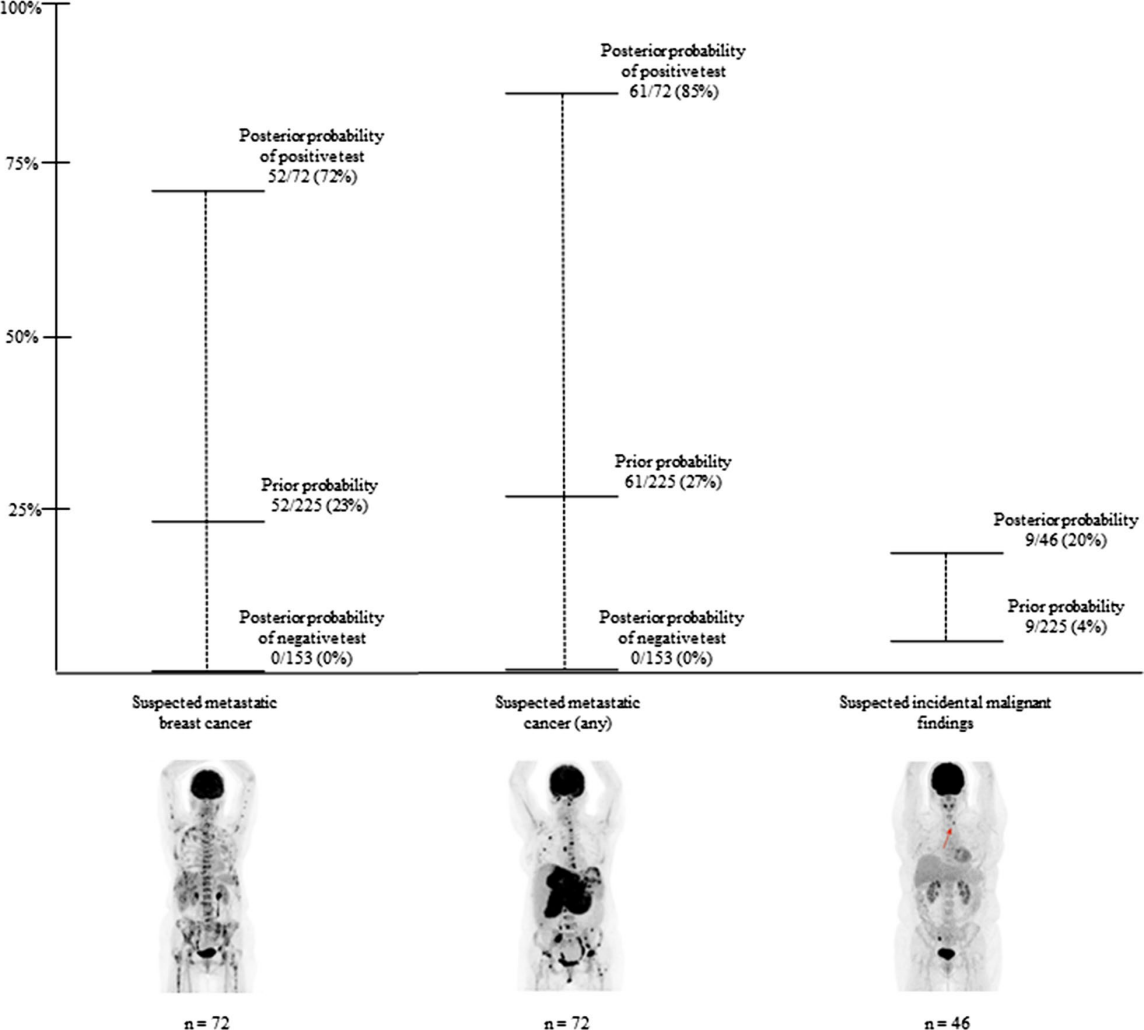
Prior and posterior probabilities for metastatic breast cancer, any metastatic cancer, and incidental malignancies according to [^18^F]FDG-PET/CT

A positive [^18^F]FDG-PET/CT provided a marked additional probability of disease, and a negative test was able to rule out distant metastasis (Fig. 2). But for incidental findings, the positive probability of positive test gave a rise in probability for synchronous cancer from 4 to 20%, only.

**Fig. 3:**
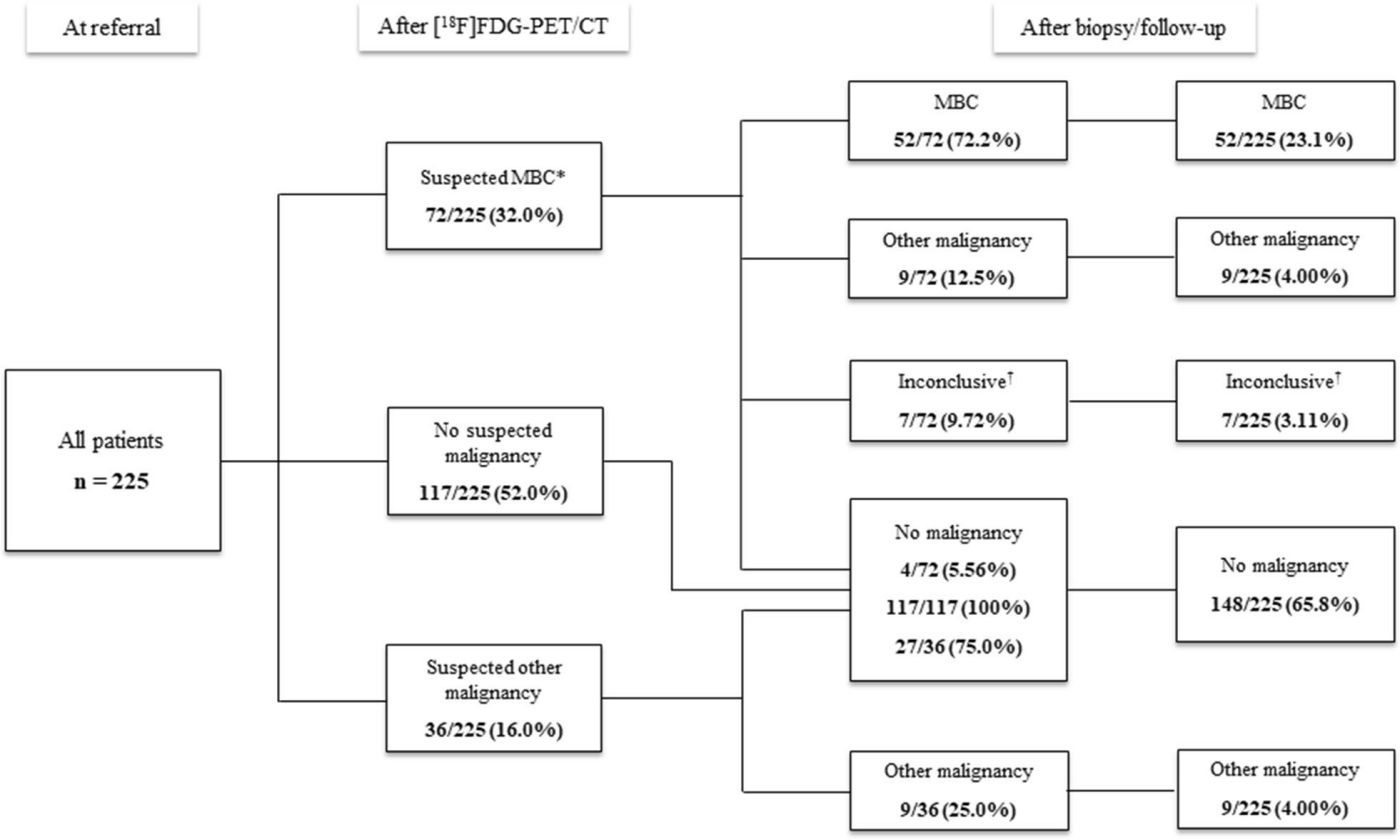
Illustration of the pathway for 225 patients after [^18^F]FDG-PET/CT and biopsy/follow-up, reflecting the results of the scan and final diagnostic workup. *Footnotes*: Metastatic breast cancer (MBC). *Ten patients suspected of MBC were also suspected of incidental findings. ^†^A final diagnosis was not obtained due to an impossible biopsy procedure or the result of the biopsy being inconclusive

Figure 3 illustrates the pathway for patients after [^18^F]FDG-PET/CT and biopsy/follow-up, reflecting the scan and final diagnostic workup results. [^18^F]FDG-PET/CT was positive for MBC in 72 patients (32.0%), and the biopsy confirmed MBC in 52 of those (72.2%), while it revealed metastases other than MBC in another nine patients (12.5%). A final diagnosis could not be confirmed by biopsy in seven patients since it was impossible to obtain a biopsy, or the biopsy result was inconclusive; all inconclusive results were suspected metastatic lesions in the lung. Regular follow-up revealed one case of distant metastasis of malignant melanoma. This case was diagnosed after progression of the suspected lesion (at 44 months), render it possible to obtain a biopsy. No instances of MBC appeared within the 6-month follow-up. [^18^F]FDG-PET/CT suggested other malignancies than MBC in 36 patients (16.0%), of which another cancer was diagnosed by biopsy in nine (25.0%). Finally, [^18^F]FDG-PET/CT detected no malignancy in the remaining 117 patients (52.0%).

Forty-seven incidental findings suspected of malignancy were detected by [^18^F]FDG-PET/CT in 46 of 225 patients (20.4%). Of those, [^18^F]FDG-PET/CT was negative for MBC in 36 patients, and in ten patients, [^18^F]FDG-PET/CT was positive for MBC, and suspicion was raised for another synchronous cancer.

**Fig. 4:**
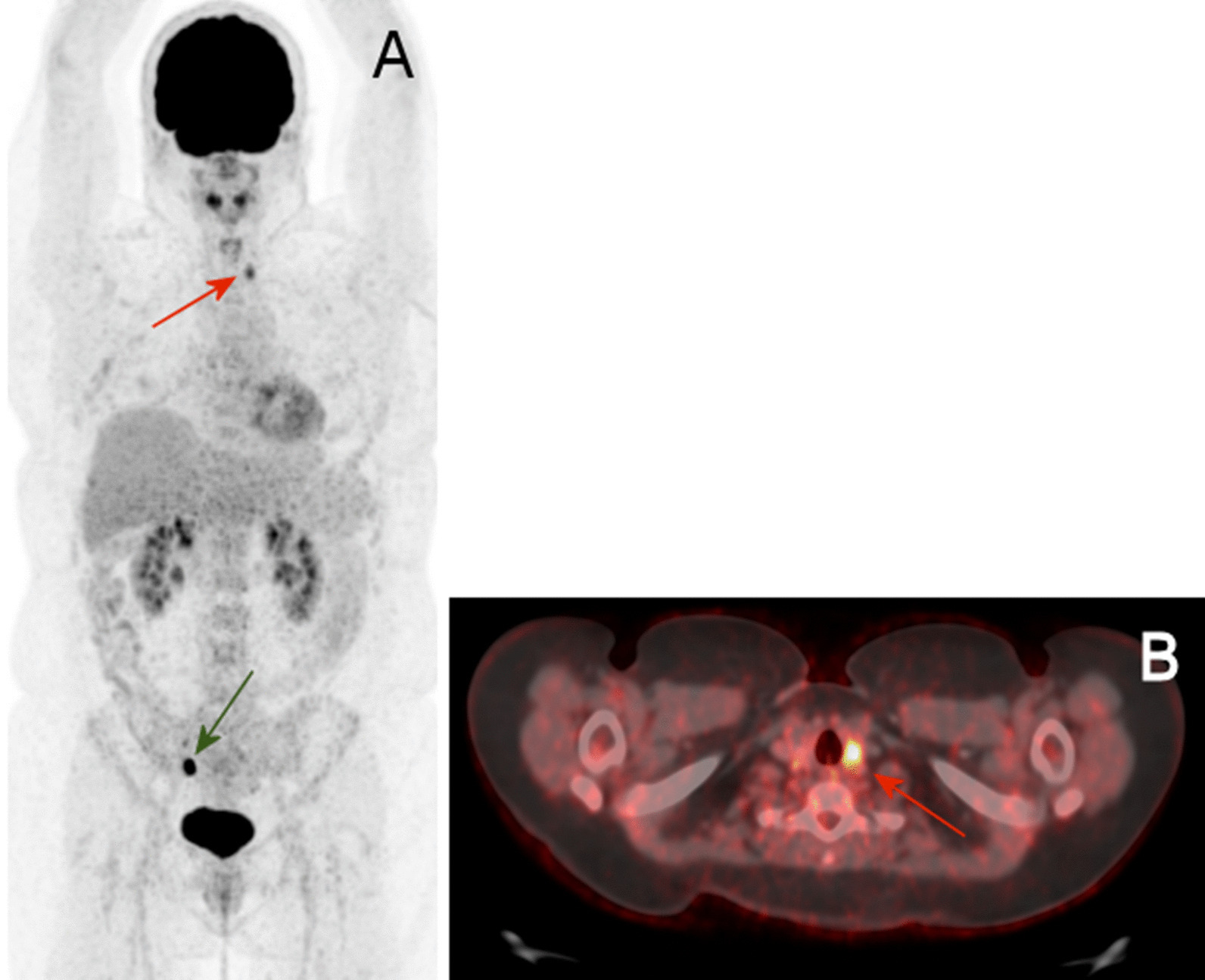
[^18^F]FDG-PET/CT in a 40-year-old woman with previous breast cancer who underwent [^18^F]FDG-PET/CT due to bone pain. [^18^F]FDG-PET/CT showed incidental focal [^18^F]FDG-uptake in the thyroid gland that revealed papillary thyroid carcinoma stage T2N1M0. A: Maximum intensity projection and B: axial section of the neck region of [^18^F]FDG-PET/CT. The red arrows show the incidental focal uptake in the left thyroid gland, and the green arrow shows incidental benign physiologic uptake in the right ovary

Details on examinations and final diagnosis are presented in Table 3. Additional examinations due to false-positive findings took place in 37/225 (16.4%) patients. The most common suspicion of malignancy was focal [^18^F]FDG-uptake in the colon (*n* = 12) or the thyroid gland (*n* = 12). After further examinations, a diagnostic workup revealed other malignancies in nine of 46 patients (19.6%)—an example of this is seen in Fig. 4.

**Table 3:** Examinations performed after suspected incidental malignancies on [^18^F]FDG-PET/CT in women with confirmed and not confirmed findings (*n* = 46)

Suspected other malignancies*			
Confirmed other malignancies	*n*	Not confirmed other malignancies ^†^	*n*
	9 (4.00%)		37 (16.4%)
*Sites of suspected lesions*			
Thyroid cancer	1	Colon or upper gastrointestinal	15
Lung cancer	1	Thyroid	11
Skin cancer	1	Head and neck	4
New breast cancer	1	Uro-gynecological	2
Peritoneal cancer	1	Lung	2
Kidney cancer	1	Mesenteric lymph node	1
Malignant melanoma	1	Muscle (abdominal wall)	1
Hematological cancer	1	Breast	1
		Kidney	1
*Clinical consequences*			
Biopsy^¤^	8	Clinical examinations	16
General anesthesia	7	Ultrasound	16
Other surgery	4	Endoscopy (colon or ventricular)	14
Follow-up CT or [^18^F]FDG- PET/CT	4	Biopsy^¤^	12
Blood tests	4	Follow-up ultrasound	9
Hospital admission	3	Laryngoscopy or cystoscopy	4
Clinical examinations	3	Follow-up CT or [^18^F]FDG-PET/CT	4
Medical treatment	3	General anesthesia	3
Thyroidectomy	2	Hospital admission	3
Ultrasound	2	Other surgery	2
Bone marrow examination	1	Blood tests	2
Cryoablation	1	MRI	1
Renography	1	Endobronchial ultra sound	1
Laryngoscopy	1	Thyroidectomy	1
		CT urography	1
		Gynecological examination	1
		Severe complications	1

^*^Ten patients suspected of incidental malignant findings were also suspected of MBC

^†^One patient had two additional incidental malignant findings

^¤^Including fine-needle aspiration in suspected lesions in the thyroid gland

Further examinations of 10 benign incidental findings included: 8 clinical examinations, two scans (CT and MRI), one endoscopy, and two biopsies. In 3 of 10 (30%) patients, further examinations revealed benign diseases with a need for treatment or further follow-up: two patients were diagnosed with rheumatic polymyalgia with the need for steroid treatment and one with cholecystitis with the need for gallbladder stone removal.

### Accuracy

The sensitivity for diagnosing distant metastases was 1.00 (95% confidence interval: 0.93–1.0), specificity 0.88 (0.82–0.92), the positive predictive value 0.71 (0.59–0.81), the negative predictive value 1.00 (0.98–1.00), the accuracy 0.91 (0.86–0.94), and the AUC-ROC 0.98 (0.97–0.99) as seen in Additional file 1: Fig. 1. For CE-CT, the sensitivity was 0.96 (0.87–1.00), specificity 0.88 (0.83–0.93), the positive predictive value 0.71 (0.59–0.82), the negative predictive value 0.99 (0.95–1.00), the accuracy 0.90 (0.86–0.94), and the AUC-ROC 0.95 (0.92–0.98, Additional file 1: Fig. 1). None of the women with a negative [^18^F]FDG-PET/CT for MBC experienced distant recurrence within 6 months follow-up; hence, all were categorized as true-negative. False-positives among women suspected of MBC were due to the diagnosis of other metastatic malignancies in nine of 20 patients where metastatic lesions detected on [^18^F]FDG-PET/CT were considered from breast cancer.

## Discussion

Approximately, one in four women suspected of distant recurrence on [^18^F]FDG-PET/CT were diagnosed with MBC by biopsy in a daily clinical setting. The posterior probability of a positive [^18^F]FDG-PET/CT for any metastatic cancer was 85%; however, examinations of the false-positive findings were performed in one of six. Incidental malignant findings other than MBC were suspected by [^18^F]FDG-PET/CT in every five women, and further examinations revealed other cancer diseases in approximately one-fifth of them. Sensitivity, specificity, and AUC-ROC of [^18^F]FDG-PET/CT were 1.00, 0.88, and 0.98, respectively.

Clinical benefits of [^18^F]FDG-PET/CT in women suspected of recurrent breast cancer are reflected by the prior and posterior probabilities of being diagnosed with metastatic cancer. However, the clinical drawbacks are the high false-positive rate that implies a risk for referral for supplemental examinations of which the patient could have been spared. For the latter, downplaying the harmfulness of such findings, e.g., in the colon or lungs, and carefully weighing the communication to the patient seems to be of great importance. Hesitancy to invasive procedures and watchful waiting should be considered instead of not referring to [^18^F]FDG-PET/CT.

In total, 18 other cancers were detected by [^18^F]FDG-PET/CT. In nine patients, the origin of the detected metastases was suggested to be breast cancer, and [^18^F]FDG-PET/CT suggested synchronous cancer in the remaining 9 of 225 patients (4%). This number corresponds well with the 3.88% of incidental findings in a population of high-risk primary breast cancer in the same institution and time [10]. Other studies on different cancers have also found between 1–8% of additional malignancies at [^18^F]FDG-PET/CT [9, 15–18].

Colon and the thyroid gland were the most common lesions with [^18^F]FDG-uptake that needed further examination. None of the patients (0 of 12) in this cohort were diagnosed with colorectal cancer, but one out of 12 patients (8.3%) with focal [^18^F]FDG-uptake in the thyroid gland was diagnosed with thyroid cancer. Our findings are based on small numbers but correspond well with the approximately 8% malignancy rates found in other studies of focal [^18^F]FDG-uptake in the thyroid gland [19, 20].

This study confirms the high diagnostic accuracy of [^18^F]FDG-PET/CT in examining suspected recurrent breast cancer reported in previous studies [4, 21]. We found distant metastases in 52 of 225 (23.1%) women with clinically suspected recurrent breast cancer referred for [^18^F]FDG-PET/CT, which is in line with an earlier study in our institution [4] with 22 of 100 (22.0%) diagnosed with distant metastases. The sensitivity of 1.00 was equal to that in the earlier study, but we found a slightly lower specificity of 0.88 (0.82–0.92) compared with 0.91 (0.83–0.96) earlier. The higher rate of false-positive findings was related to the diagnosis of other malignancies in nine patients where metastatic lesions detected on [^18^F]FDG-PET/CT were considered to be from breast cancer. Further, another seven patients had suspected metastatic lesions with inconclusive biopsy results and no progression of lesions during follow-up, and hence, they were considered false-positive.

Patients had [^18^F]FDG-PET/CT performed with diagnostic contrast-enhanced CT, which exposes them to a radiation dose of 12–16 mSv. The excess radiation dose for women in this study was restricted compared with the traditional examination program of CE-CT and bone scintigraphy, with a combined radiation dose of approximately 14 mSv.

A major strength in this study is the prospective design of daily clinical practice with confirmatory biopsies for patients diagnosed with MBC. Further, the overall clinical impact of the false-positive rate and accuracy was evaluated. The study was registered at ClinicalTrials.gov with an enrolment of patients with high-risk primary breast cancer. This population has been described elsewhere as the clinical impact of [^18^F]FDG-PET/CT differs between patients with high-risk primary breast cancer and patients suspected of recurrent breast cancer [10]. Another strength is that we managed to reproduce and validate the diagnostic accuracy of an earlier study [4]. Reproducibility and transparency are key parts of the scientific path and are crucial for implementing research results in clinical guidelines [22–25]. Despite excellent sensitivity and high specificity, [^18^F]FDG-PET/CT remains to be implemented in clinical guidelines as the diagnostic modality for recurrent breast cancer.

A notable limitation in this single-center validation study was the assessment of CE-CT scans due to the availability of clinically used bookmarks and knowledge of coming scans, all of which were accessible by the radiologist. This provided the radiologist with information on metastatic lesions, and the accuracy of CE-CT might therefore be overestimated in this study.

[^18^F]FDG-PET/CT has already been implemented as a stand-alone examination in suspected recurrent breast cancer in our institution. Although we acknowledge the impact of supplemental examinations and the shortage of [^18^F]FDG-PET/CT scanners in other countries, we wish to encourage [^18^F]FDG-PET/CT to be implemented in clinical guidelines for diagnosing recurrent breast cancer.

Clinical follow-up after early-stage breast cancer comprises mammography, and no recommendations exist on whole-body examinations, particularly not for high-risk patients. However, these recommendations arose from a former time with less advanced image techniques and less efficacious treatments for MBC [7]. Since patients in this study had very widespread disease with more than five metastatic lesions, a perspective for future studies could be to explore the impact of [^18^F]FDG-PET/CT as follow-up after high-risk early-stage breast cancer, i.e., patients treated for locally advanced breast cancer, extensive lymph node involvement in the axilla, triple-negative, and HER2 positive breast cancer. Such studies should be designed to address the false-positive rates and cost–benefit analyses as well. [^18^F]FDG-PET/CT has high sensitivity and could be the modality to detect oligometastatic disease, which could translate into potential patient benefit.

The sum of prognostic markers of primary breast cancer, time since primary diagnosis, and symptoms leading to referral for suspected recurrent breast cancer add to the patient's prior probability of suffering from distant recurrent breast cancer. The variety of symptoms leading to the suspicion of recurrent breast cancer and management of incidental findings suspected of malignancy on FDG-PET/CT call for experienced physicians in breast cancer with a multimodality approach as suggested by the European Society of Breast Cancer Specialists (EUSOMA) [26].

## Conclusions

In conclusion, [^18^F]FDG-PET/CT provided a high posterior probability of a positive test, and a negative test was able to rule out distant metastases in women with clinically suspected recurrent breast cancer. One-fifth of patients examined for incidental findings detected at [^18^F]FDG-PET/CT were diagnosed with clinically relevant conditions. Further examinations of false-positive incidental findings in one of six women should be weighed against the high accuracy for diagnosing metastatic breast cancer.

## Supplementary Information

**Additional file 1**: Fig. 1. Receiver operating characteristic (ROC) curve and area-under-the-receiver operating curve (AUC-ROC) derived from [18F]FDG-PET/CT and CE-CT for the detection of distant metastasis in 225 women suspected of first distant recurrent breast cancer

**Additional file 2.** Characteristics of the primary tumor of 225 women suspected of first distant recurrent breast cancer, 2017–2019.

## Data Availability

The dataset supporting the conclusions of this article is available from the corresponding author on reasonable request.

## Abbreviations

AUC-ROC: Area-under-the-receiver operating curve
CE-CT: Contrast-enhanced CT
CI: Confidence interval
ER: Estrogen receptor
[^18^F]FDG-PET/CT: [^18^F]-Fluorodeoxyglucose-positron emission tomography/computed tomography
HER2: Human epidermal growth receptor 2
MBC: Metastatic breast cancer
MRI: Magnetic resonance imaging
RedCap: Research electronic data capture
