# Supplementary figures and images for: Benefits and harms of implementing [18F]FDG-PET/CT for diagnosing recurrent breast cancer: a prospective clinical study
Source: EJNMMI Res. 2021 Sep 22;11:93. doi: 10.1186/s13550-021-00833-3 (PMC8458550; doi:10.1186/s13550-021-00833-3)

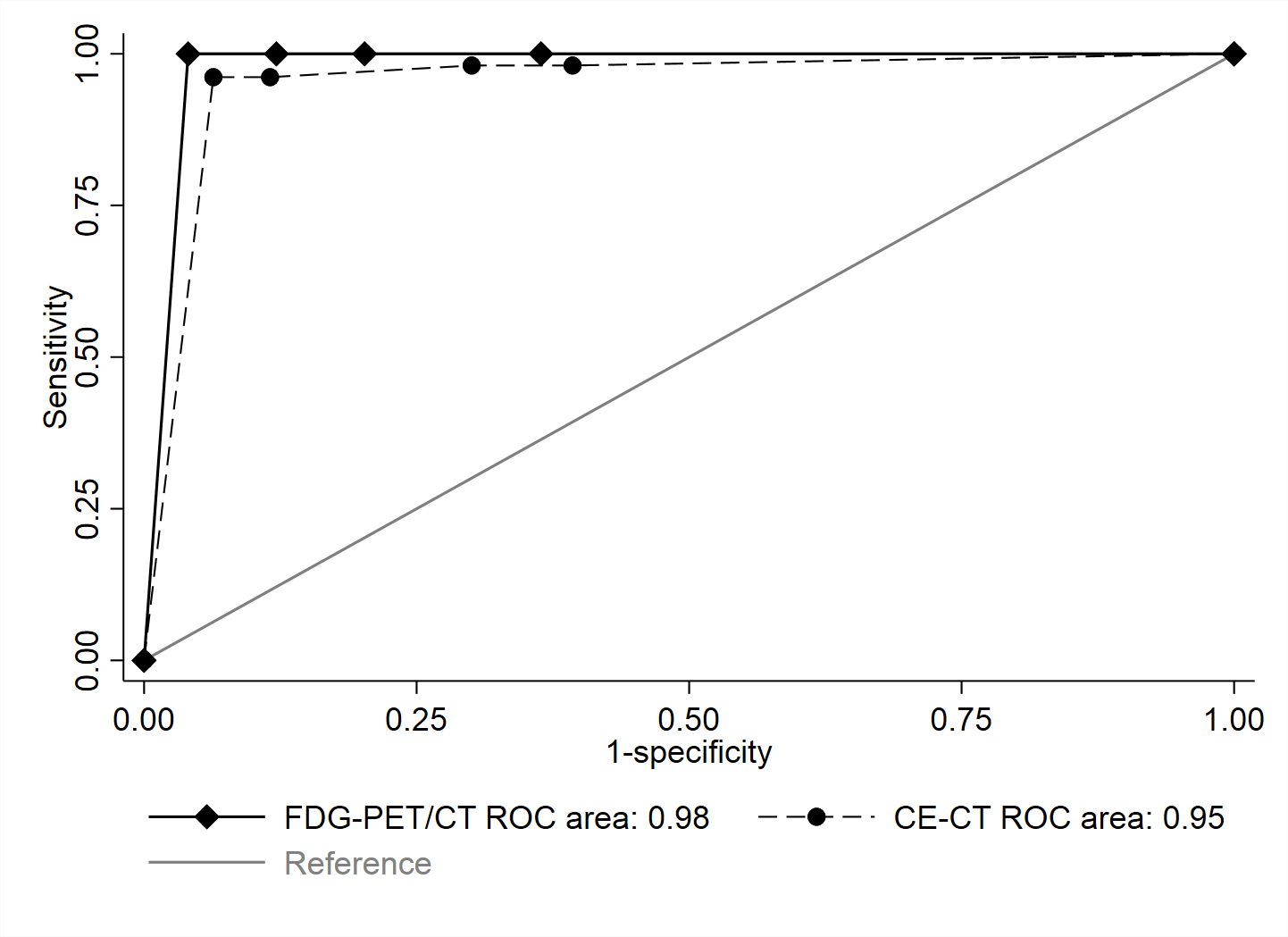

Supplement: Supplementary file 1 — Additional file 1: Fig. 1. Receiver operating characteristic (ROC) curve and area-under-the-receiver operating curve (AUC-ROC) derived from [18F]FDG-PET/CT and CE-CT for the detection of distant metastasis in 225 women suspected of first distant recurrent breast cancer [file 13550_2021_833_MOESM1_ESM.tif]
