# Supplementary material for: Benefits and harms of implementing [18F]FDG-PET/CT for diagnosing recurrent breast cancer: a prospective clinical study
Source: EJNMMI Res. 2021 Sep 22;11:93. doi: 10.1186/s13550-021-00833-3 (PMC8458550; doi:10.1186/s13550-021-00833-3)
Supplement: Supplementary file 2 — Additional file 2. Characteristics of the primary tumor of 225 women suspected of first distant recurrent breast cancer, 2017–2019. [file 13550_2021_833_MOESM2_ESM.docx]

## Supplemental Table 1

Characteristics of the primary tumor of 225 women suspected of first distant recurrent breast cancer, 2017-2019.

|  | All patients |
| --- | --- |
| Characteristics | N (%) |
| **Bilateral disease** |  |
| Yes | 6 (2.67) |
| No | 219 (97.3) |
| **Type of surgery** |  |
| Mastectomy | 96 (42.7) |
| Breast-conserving* | 128 (56.9) |
| Other | 1 (0.44) |
| **Tumor size** |  |
| ≤10 mm | 43 (19.1) |
| 11-20 mm | 107 (47.6) |
| 21-50 mm | 59 (26.2) |
| ≥50 mm | 7 (3.11) |
| Unknown | 9 (4.00) |
| **Lymph node involvement** |  |
| 0 | 122 (54.2) |
| 1-3 | 68 (30.2) |
| 4-9 | 13 (5.78) |
| ≥ 10 | 13 (5.78) |
| Unknown | 9 (4.00) |
| **Histology** |  |
| Ductal carcinoma | 171 (76.0) |
| Lobular carcinoma | 18 (8.00) |
| Carcinoma NOS | 5 (2.22) |
| Other | 28 (12.4) |
| Unknown | 3 (1.33) |
| **Grade of malignancy** |  |
| I | 55 (24.4) |
| II | 75 (33.3) |
| III | 46 (20.4 |
| Unknown/Not graded | 49 (21.8) |
| **ER status** |  |
| Negative (0%) | 45 (20.0) |
| Positive (1-9%) | 1 (0.44) |
| Positive (10-100%) | 171 (76.0) |
| Unknown | 8 (3.56) |
| **HER-2 status** |  |
| Normal | 164 (72.9) |
| Positive | 23 (10.2) |
| Unknown | 38 (16.9) |
| **No. of local recurrence or other breast cancer** |  |
| 0 | 157 (69.8) |
| 1 | 60 (26.7) |
| 2 | 8 (3.56) |
| **Medical treatment for early breast cancer** |  |
| Neoadjuvant +/- adjuvant treament | 17 (7.56) |
| Adjuvant treatment | 152 (67.5) |
| No medical treatment | 51 (22.7) |
| Unknown | 5 (2.22) |
| **Total** | **225 (100)** |
|  |  |

^Estrogen receptor (ER), Human Epidermal Growth Factor receptor 2 (HER-2)^

^*Women who underwent breast-conserving surgery were considered for adjuvant radiotherapy as suggested in clinical guidelines.^
